# Molecular and Serological Characterization of the SARS-CoV-2 Delta Variant in Bangladesh in 2021

**DOI:** 10.3390/v13112310

**Published:** 2021-11-19

**Authors:** Asish Kumar Ghosh, Marco Kaiser, Md. Maruf Ahmed Molla, Tasnim Nafisa, Mahmuda Yeasmin, Rifat Hossain Ratul, Md. Mohiuddin Sharif, Arifa Akram, Nur Hosen, Rashid Mamunur, Md. Robed Amin, Alimul Islam, Md. Ehsanul Hoque, Olfert Landt, Simon D. Lytton

**Affiliations:** 1 Department of Virology, Dhaka Medical College Hospital, Dhaka 1000, Bangladesh; asish127kumar@gmail.com (A.K.G.); dr.ratulrifat@gmail.com (R.H.R.); mohiuddinsharif@pircc.org (M.M.S.); robedamin@yahoo.com (M.R.A.); 2 GenExpress Gesellschaft für Proteindesign GmbH, Eresburgstraße 22-23 D, 12103 Berlin, Germany; kaiser@genexpress.de; 3 National Institute of Laboratory Medicine and Referral Center, Sher E-Bangla Nagar, Dhaka 1207, Bangladesh; maruf063@gmail.com (M.M.A.M.); nafisahf3@gmail.com (T.N.); shumi.yeasmin@gmail.com (M.Y.); drbarna43@gmail.com (A.A.); nurhosen568@gmail.com (N.H.); nilmrc@ld.dghs.gov.bd (M.E.H.); 4 Bangladesh Institute Tropical Infectious Disease (BITID), Fouzderhat, Chittagong 4317, Bangladesh; mamunurdr30@gmail.com; 5 Department of Microbiology and Hygiene, Faculty of Veterinary Science, Bangladesh Agricultural University, Mymensingh 2202, Bangladesh; alimul.vmh@bau.edu.bd; 6 TIB Molbiol GmbH, Eresburgstraße 22-23, 12103 Berlin, Germany; OLandt@tib-molbiol.de; 7 SeraDiaLogistics, 81545 Munich, Germany

**Keywords:** SARS-CoV-2, delta variant, Bangladesh, anti-S-protein IgG, anti-N-protein IgG, amino acid mutations, single nucleotide polymorphism, RT-PCR, VirSNiP, sequencing

## Abstract

Novel SARS-CoV-2 variants are emerging at an alarming rate. The delta variant and other variants of concern (VoC) carry spike (S)-protein mutations, which have the potential to evade protective immunity, to trigger break-through infections after COVID-19 vaccination, and to propagate future waves of COVID-19 pandemic. To identify SARS CoV-2 variants in Bangladesh, patients who are RT-PCR-positive for COVID-19 infections in Dhaka were screened by a RT-PCR melting curve analysis for spike protein mutations. To assess the anti-SARS CoV-2 antibody responses, the levels of the anti-S -proteins IgA and IgG and the anti-N-protein IgG were measured by ELISA. Of a total of 36 RT-PCR positive samples (75%), 27 were identified as delta variants, with one carrying an additional Q677H mutation and two with single nucleotide substitutions at position 23029 (compared to Wuhan-Hu-1 reference NC 045512) in the genome sequence. Three (8.3%) were identified as beta variants, two (5.5%) were identified as alpha variants, three (8.3%) were identified as having a B.1.1.318 lineage, and one sample was identified as an eta variant (B.1.525) carrying an additional V687L mutation. The trend of higher viral load (lower Cp values) among delta variants than in the alpha and beta variants was of borderline statistical significance (*p* = 0.045). Prospective studies with larger Bangladeshi cohorts are warranted to confirm the emergence of S-protein mutations and their association with antibody response in natural infection and potential breakthrough in vaccinated subjects.

## 1. Introduction

The severe acute respiratory syndrome coronavirus 2 (SARS-CoV-2), first identified in Wuhan province, China, in December 2019, has become responsible for the Corona-virus disease 2019 (COVID-19) pandemic, causing over five million reported COVID-19 deaths worldwide, of which 25% are reported from the United States and India [1]. The global impact of COVID-19 on human health and human mobility is evidenced by the disruption in social, psychological, and economic well-being in almost all countries of the world. The swift response of government–industry partnerships in the development, clinical evaluation, and fast-track emergency use authorization of SARS-CoV-2 vaccines in 2020 was followed by a coordinated effort in vaccine manufacturing and distribution [2,3,4]. Six billion dosages of vaccines were administered between January 2021 and the date of this manuscript submission [1]. The expectation of “reaching herd immunity” and preventing the transmission of SARS-CoV-2 viruses through vaccination is thwarted by asymmetries in vaccine supply between high- and low-income countries and the lack of resources for the frozen storage and cold-chain distribution of mRNA vaccines in Africa and Asia [5]. In low-income countries with high population density, government-mandated lockdown and social distancing orders are often impractical to implement [6,7]. In high-income countries, political lobbies and social media have spread confusion and misinformation over vaccines and face covering [8]. These socioeconomic and behavioral factors have affected the rates of SARS-CoV-2 transmission and community-spread of SARS CoV-2 strains predicted by non-vector borne susceptible, infected, and recovered (SIR) models [9].

The SARS-CoV-2 reference sequence has undergone genetic changes since 2019, raising speculation that variants are emerging capable of immune evasion and resisting treatment and vaccines [10,11,12,13]. The Global Initiative on Sharing All Influenza Data (GISAD) introduced a nomenclature system to assign SARS-CoV2 mutations according to clades [14]. Based on the SARS-CoV-2 genome sequences, a software tool termed Phylogenetic Assignment of Named Global Outbreak (PANGO) Lineages was developed to classify the genetic lineages [15,16,17]. The SARS CoV-2 variant classifications and definitions were initially introduced as variants of concern (VoC) or variant of interest (VoI) and subsequently expanded to include variants under investigation (VUI) or variants being monitored (VBM) by the Center of Disease Control Atlanta (CDC) [18] and Public Health England (PH) [19]. The labels alpha, beta, gamma, delta, and eta were designated by the world health organization (WHO) [20].

The commercially approved COVID-19 vaccines and many of the COVID-19 vaccine candidates currently under development are derived from the first available SARS-CoV-2 S-protein angiotensin converting enzyme-2 (ACE-2) receptor sequences of Wuhan genome NC045512.2 [10,11].

The question of whether not the delta variant carries S-protein amino acid mutations, which contribute to greater transmissibility than alpha or beta variants and hinder vaccine efficiency, is a topic of major concern [21,22]. The effectiveness of COVID-19 vaccines BNT162b2 or ChAdOx1 nCoV-19 in the United Kingdom were evaluated retrospectively in 14,387 alpha variant infections and 4272 delta variant infections. With BNT162b2 vaccines, the effectiveness of two doses was 93.7% among persons with the alpha variant and 88% among persons with the delta variant [23]. With ChAdOx1 nCoV-19, the effectiveness of two doses was 74.5% among persons with the alpha variant and 67% among those with the delta variant.

On the 27 January 2021, the Oxford AstraZeneca vaccine manufactured at the Serum Institute of India was introduced in Dhaka, and to date, 13 million out of the 164 million Bangladeshi population have received their second vaccine dose [24]. In March 2021, routine SARS CoV-2 RT-PCR screening of health care workers by the Child Health Research Foundation (CHRF) in Dhaka confirmed the PANGO lineage B.1.351 (beta variant) by genome sequencing [25]. Curiously, two of the four B.1.351 reported cases, without previous COVID-19 history, were breakthrough infections with SARS-CoV-2 RT-PCR positivity on day 34 and day 36 after the first dose of the ChAdOx1 vaccine [25]. In March 2021, the alpha variant positivity rate was 53% [26]. During April and May 2021, the beta variant was detected among 90% of the SARS CoV-2 RT-PCR-positive cases, and in May and June 2021, the delta variant was first reported in Dhaka city [26].

Our single-center cross-sectional survey of SARS-CoV-2 variants in Dhaka has screened for the spike protein mutations by single nucleotide polymorphism RT-PCR (VirSNiP test) and confirmed the mutations by sequencing. The titers of anti-spike protein and anti-nucleocapsid protein antibodies were measured by ELISA. In this study, we detect SARS CoV-2 variants circulating in unvaccinated subjects and show that the delta variant is predominant in Dhaka city during the two-week period between 26 May and 6 June 2021. The limited sample size is not sufficient to assess the statistical significance of the antibody responses found among patients infected with SARS CoV-2 variants carrying rare amino acid mutations in the spike protein sequence. The findings underscore the importance of monitoring the emergence of SARS-CoV-2 variants during natural SARS-CoV-2 infection in a low-income country with 3 percent of its population receiving COVID-19 vaccines.

## 2. Materials and Methods

### 2.1. Study Design and Sample Collections

This study is a cross-sectional single-center study on thirty-six Bangladeshi adults testing RT-PCR SARS-CoV-2 positive with symptomatic COVID-19 between 26 May 2021 and 6 June 2021 at Dhaka Medical College. All cases were randomly selected for the survey of SARS CoV-2 variants and had not received any COVID-19 vaccination. Nasopharyngeal specimens were collected and stored at −80 °C in a virus collection and preservation medium (Khang Jian Medical Apparatus Ltd., Taizhou, China) and transported on dry ice to Germany. Serum samples matched to the date of swab collection, *n* = 36, and at follow-up on day 13 or day 14, *n* = 28, or on day 17, *n* = 1, post onset of COVID-19 symptoms (POCS) were kept at −20 °C storage in screw-tight cryopreservation vials (Greiner-bio one).

### 2.2. Quantification of Viral RNA

The viral RNA of nasopharyngeal specimens was extracted with a Magnapure 96 instrument (Roche, Mannheim, Germany) using the DNA and Viral nucleic acid kit (Roche, Penzberg, Germany). The RNA concentration in each sample was determined by reverse transcriptase polymerase chain reaction (RT-PCR) using the LightMix^®^ Modular SARS-CE assay (50-0776-96, TIB MolBiol, Berlin, Germany) and programming on 480II light cycler (Roche, Penzberg, Germany). The point at which the amplification curve crossed the vertical threshold line in the RT PCR cycle (Cp) was reported as positive if the sample Cp was less than 40.

### 2.3. Screening of Spike Protein Mutations and Sequencing

The spike protein variants H69V70del, Y144del, T478K, K417N/T, P681R/H, E484K, and N501Y were identified using a RT-PCR melting curve analysis targeting amino acid mutations. The VirSNiP SARS-CoV-2 typing assays (TibMolBiol, Cat.-No. 53-0781-96, 53-0799-96, 53-0807-96, 53-0811-96, 53-0813-96) and LightCycler^®^ Multiplex RNA Virus Master (Roche Cat.-No. 06 754 155 001) were performed following the manufacturer’s instructions. The six pairs of forward (F) and reverse (R) sequencing primers were designed according to the SARS-CoV-2 reference strain NC_045512, resulting in overlapping fragments covering 2.1 kb of the spike-gene (Supplementary Table S1). The amplification conditions were as follows: RT at 55 °C for 5 min, activation for 2 min at 95 °C, 45 cycles with 10 s at 95 °C, 20 s at 58 °C, and 30 s at 72 °C for product amplification. The amplified products with 411–501 bp were purified with NucleoSpin Gel and PCR clean-up (Macherey-Nagel, Düren, Germany), sequenced using the BigDye version 3.1 cycle sequencing kit (Thermo Fisher Scientific Life Technologies GmbH, Waltham, MA, USA), and subjected to an automated sequence analysis using the SeqStudio Genetic Analyser (Thermo Fisher Scientific Inc., Waltham, MA, USA).

### 2.4. Bioinformatics

Sequence and phylogenetic analyses were conducted using software program MEGA version X. The derived sequences were compared with the data from Global Initiative on Sharing All Influenza Data (GISAID) database and analyzed according to the phylogenetic assignment of named global outbreak (PANGO) lineages [27].

### 2.5. Assessment of Anti-SARS CoV-2 Antibodies

The anti-SARS-CoV-2 spike (S) protein IgA and the anti-SARS-CoV-2 S-protein IgG Quanti vac ELISA (EUROIMMUN Medizinische Labordiagnostika AG, Germany) were measured according to the manufacturer’s instructions. The positive IgA reactivity was reported as OD450nm − OD620nm > 0.22 and the positive anti-S-protein IgG > 11 RU/mL IgG. The positive anti-nucleocapsid (N)-protein IgG reactivity was according to the recommended cut-off value of NovaTec units (NTU) > 10 for positive results (Novatec Diagnostics, Dietzenbach, Germany); NTU = X × 10/QC, where X = OD450 nm − OD620 nm of the test sample and QC = OD450 nm − OD620 nm of the quality control equivocal serum sample. E > 10 NTU. All ELISA measurements were performed on a Multiskan 96 well reader (Lot 357−706872, Thermofischer Scientific Life Technologies, Darmstadt, Germany).

### 2.6. Statistical Analysis

The Cp values, the levels of the anti-SARS CoV-2 S-proteins IgA and IgG, and the levels of the anti-N-protein IgG are presented as the mean with a standard deviation and as medians with ranges. Comparisons of the values between patient groups were assessed by a nonparametric Mann–Whitney sum rank test or Chi-square analysis. A *p* value of ≤ 0.05 was considered statistically significant with differences between independent groups. Correlation analyses were calculated with the Spearman’s rank correlation coefficients. The correlation coefficients r > 0.4 or r < −0.4 with significance at *p* < 0.05 were considered strongly positive or strong negative associations, respectively. Statistical analysis was performed with MedCalc version 14 for Windows (MedCalc Software, Mariakerke, Belgium).

## 3. Results

### 3.1. SARS CoV-2 Variants

**Figure 1:**
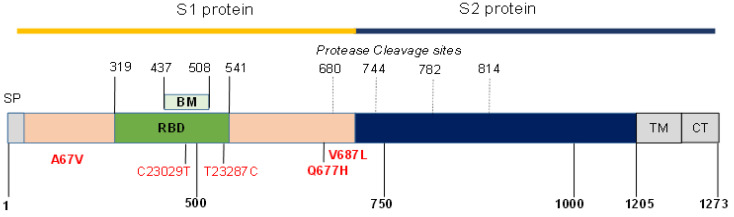
Position map of S-protein mutations in SARS CoV-2. The position of rare amino acid mutations and the single nucleotide exchanges in the NC_045512 Wuhan-Hu-1 genome sequence identified in this study are highlighted in red. The GSAID accession numbers are listed in Table 2.

Of the 36 (14%) RT-PCR-positive cases, 5 belonged to known variants: two alpha (H69V70del and N501Y) and three beta (K417N, E484K, and N501Y), designated as group 1 (Table 1). Of a total 36 RT-PCR-positive Dhaka COVID-19 cases, 27 (75%) were identified as delta variants. Of the 27, 24 had amino acid substitutions G142D, T478K, and P681R, matching the known delta variants in India, and were assigned to group 2. The other 3 of the 27 delta variants had aberrant melting curves in the VirSNiP SARS-CoV-2 typing assay and were assigned after sequencing to the group 3 “delta plus” (Table 1). The sequencing of spike gene fragments from these three delta plus variants showed one carrying an additional Q677H mutation and the other two car-rying single nucleotide substitutions C23045T compared with the reference wild-type Hunan SARS-CoV-2 strain NC-045512 in the genome sequence (Figure 1 and Table 2). The remaining 4 of the 36 (11%) RT-PCR-positive cases in group 4 were other variants: three were matched to the PANGO lineage B.1.1.318, of which two carried nucleotide substitution T23287C without amino acid change (Table 2). The variant of interest (VOI), the eta variant belonging to the B.1.525 lineage, revealed a novel amino acid substitution V687L in addition to A67V and Q677H (Table 2).

### 3.2. Bangladeshi COVID-19 Patient Characteristics

All thirty six of the enrolled SARS-CoV-2 RT-PCR patients were unvaccinated adults with symptomatic COVID-19 in the Dhaka district. The group 1 alpha or beta SARS-CoV-2 variants presented clinical symptoms at similar frequency to the clinical symptoms of group 2 delta SARS-CoV-2 variants, with the exception of loss of smell or taste, which was reported at significantly higher frequencies among group 1 than group 2.

### 3.3. SARS CoV-2 Variant Viral Loads

**Figure 2:**
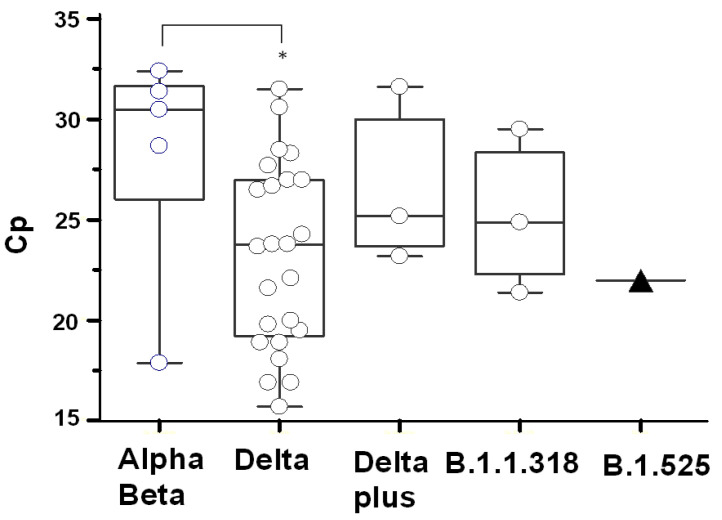
Viral loads for the SARS-CoV-2 variants. The viral RNA in each nasopharyngeal specimen is quantified by the crossing point (Cp) on the RT-PCR amplification curve. The median, 95% CI, and range of Cp values for the SARS-CoV-2 variants and B.1.1.318 PANGO lineage are represented by box-whisker plots. The single Cp value of the eta variant belonging to the B.1.525 PANGO lineage is represented by a filled triangle.

The median Cp 24 of delta variants in group 2 is significantly lower than the median Cp 31 of alpha or beta variants in group 1 (Table 1 and Figure 2). The variants in group 3 and group 4 show a trend for lower Cp values than that of group 1, but the number of cases is insufficient to assess if the differences in viral loads are of statistical significance.

### 3.4. Antibody Responses in SARS-CoV-2 Variants

**Figure 3:**
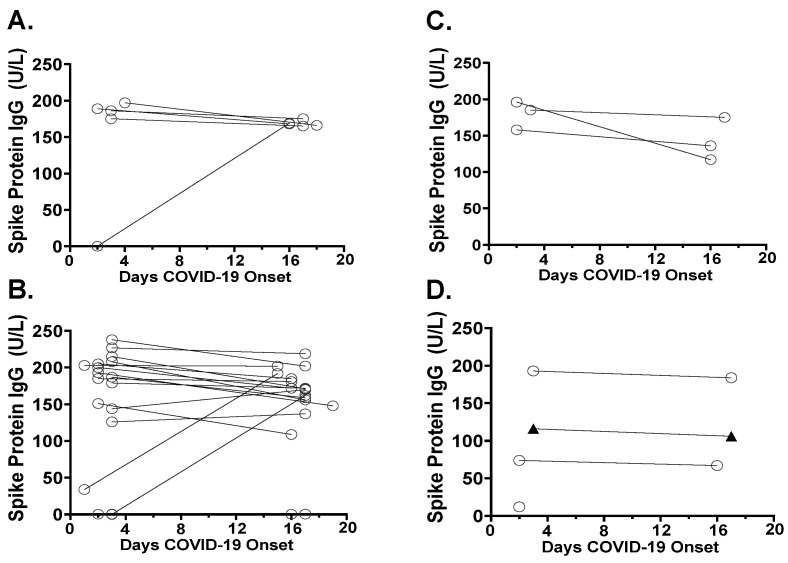
Anti-S-protein antibody levels of SARS-CoV-2 variants. The anti-S-protein antibody reactivity on day 2 or day 3 post COVID-19 onset of symptoms (POCS), and at follow up, day 16, or day 17 POCS: (**A**) alpha and beta variants, (**B**) delta variant, (**C**) delta plus variants, and (**D**) variants of the B.1.3.18 and B.1.525 PANGO lineages. Cut-off of Euroimmun Quant S-protein positivity > 11 U/L.

**Figure 4:**
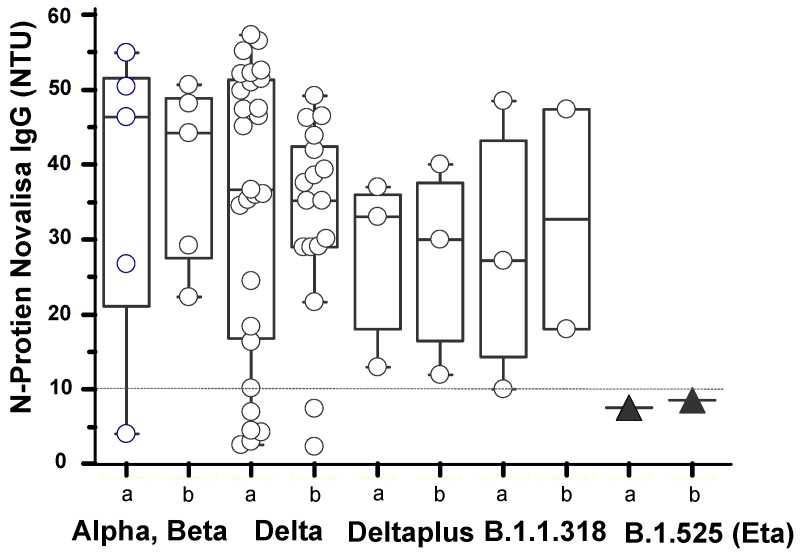
Anti-N-protein antibody levels of SARS-CoV-2 variants. The anti-N-protein IgG reactivity for patients infected with COVID-19 variants described in Figure 2; a = day 2 or day 3 POCS, and b = day 16 or day 17 PCOS. The horizontal dotted line indicates the cut-off for Novalisa positivity at >10 NTU.

Antibodies were detected against both the spike (S) protein (Figure 3) and the nucleocapsid (N) protein (Figure 4). Of the 24 (8%) delta variant cases, 2 gave the negative anti-S-protein IgG. Only 1 of the 24 delta variant cases tested negative for both anti-S-protein and anti-N-protein antibodies. The one case of eta variant B.1.525, although positive for anti-S-protein IgG, gave no detectable anti-N-protein reactivity (Figure 4). One of the three B.1.1.318 lineage cases were below the anti-S-protein cut-off on day 2 POCS (Figure 3D). Anti-S-protein antibody seroconversion (negative reactivity on day 2 and positive reactivity on days 16 or 17 POCS) was found in three cases: one beta variant (Figure 3A) and two delta variants (Figure 3B). The anti-S-protein IgA in the cases of alpha or beta variants of group 1 show a trend for higher levels than anti-S-protein IgA levels of the delta variants in group 2 (Table 1).

**Figure 5:**
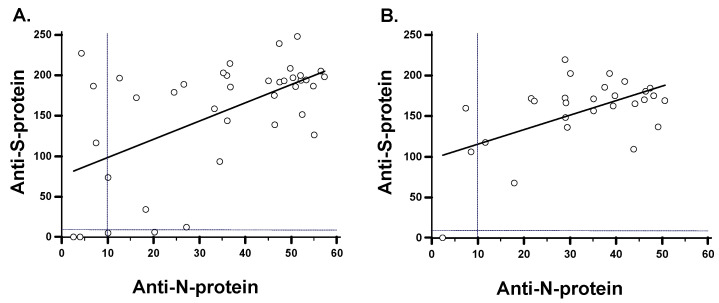
Association of anti-N-protein and anti-S-protein antibodies. The scatter plot and linear regression analyses of anti-N-protein versus anti-S-protein antibody levels. (**A**) Day 2 or day 3 PCOS; r = 2.7 (95% CI 1.7–3.75) *p* = 0.0001. (**B**) Day 16 or 17 POCS; r = 2.3 (95% CI 1.8–3.4), *p* = 0.0002. The dotted vertical and horizontal lines represent the cut-offs for positive reactivity in the respective ELISAs.

To assess the associations between the levels of anti-N-protein versus anti-S-protein antibodies, regression analyses were made on day 2 or day 3 and on day 16 or day 17 POCS. The serum anti-N-protein IgG was positively correlated with the serum anti-S-protein IgG at both time points (Figure 5). Curiously, the anti-S1-protein IgG by Euroimmun Quant ELISA consistently gave higher activities above the cut-off than the activity of the anti-N-protein IgG by Novalisa, both on day 2 or day 3 and on day 14 post onset of COVID-19 symptoms (POCS).

## 4. Discussion

The extensive genomic surveillance of SARS CoV-2 viral isolates in Bangladesh throughout the pandemic is motivated by the urgency to identify the next strains of SARS CoV-2 carrying S-protein mutations or other genetic changes [25,26,27,28,29,30,31,32,33,34]. In the first and second pandemic waves in Bangladesh, the alpha and beta variants of the B.1.1.7 and B.1.351 lineages were associated with an Ro of approximately 1.2 [27]. In the event that viral transmissibility and COVID-19 severity were to increase and threaten the collapse of the already fragile health care in the country, the government needs to rapidly implement lockdowns and to possibly develop vaccines derived from the novel SARS CoV-2 sequences [28].

The delta variant is estimated to have emerged in May 2021 in Bangladesh [26,33]. Our findings of 75% delta variant among COVID-19 patients testing SARS CoV-2 RT-PCR positive between 26 May 2021 and 6 June 2021 strongly indicate that the delta variant is well-established in Dhaka city at the time of this study and likely to be the predominant variant in Bangladesh. There are currently 1.4 million delta variants deposited in the GSAID database, of which 6818 carry the Q677H amino acid substitution; 291 of the Q677H delta variants are reported from Asia and one entry was from Bangladesh. In this respect, the Q677H delta variant in Dhaka city is a rare mutation. The Q677H is positioned adjacent to the S1/S2 cleavage site. Its effect on cell tropism and transmissibility is predicted by molecular modeling [34,35]. So far, there is no evidence that Q677H is dangerous in terms of conferring increased SARS-CoV-2 viral pathogenicity or COVID-19 disease severity [36,37]. The two delta variants carrying the C23029T single nucleotide substitution is of no relevance for the virus pathology. This synonymous substitution caused irregular melting temperatures of the E484K RT-PCR typing probe but the S-protein sequence is identical to the known delta variant.

The amino acid mutations T95I, Y144del, E484K, D614G, and P681H of the B.1.1.318 lineage were first reported in Maurtius in March 2021 SARS CoV-2 [38]. To date, there are three thousand B.1.1.318 sequences reported worldwide. The transmissibility of B.1.1.318 is comparable with that of the alpha variant. The eta variant belonging to the B.1.525 PANGO lineage is classified by the European CDC as a variant under investigation (VUI). The B.1.525 identified in this study carries a rare V687L mutation. The GSAID database has a total of 967 sequences carrying V687L mutations out of 3.8 million total SARS CoV-2 sequences. Only two eta entries have V687L (February 2021 Nigeria and May 2021 Indonesia). Further screening and sequencing of SARS CoV-2 viral isolates in Bangladesh is needed to assess the frequency of Q677H in the delta variant and V687L in the eta variant.

The major limitation of our study is the low number of RT-PCR-positive COVID-19 cases investigated during the brief two-week sampling period. The positive anti-S protein IgG and anti-N protein IgG on the day of RT-PCR positive nasopharyngeal swab and on day 16 or day 17 post onset of COVID-19 symptoms in 33 out of 36 COVID-19 patients indicate prior SARS CoV-2 infection. Our finding is supported by reports of high anti-S protein IgG on the day of positive swab among unvaccinated COVID-19 patients in the United Kingdom having confirmed SARS-CoV-2 re-infection [39]. On this basis, we conclude that the patients with the delta variant likely have pre-existing antibodies from either the first Bangladesh pandemic wave in April to June 2020 [29,30,31,32] or the second Bangladesh pandemic wave in March 2021, which were predominantly of the SARS CoV-2 alpha or beta variants [25,26,27,28,40].

The Euroimmun Quant ELISA detects anti-S1 protein IgG that has been calibrated to the antibody neutralizing activity assessed by the suppression of cytopathic effect on Vero cells infected with the German SARS CoV-2 strain01/2020, lineage B.3. [41]. Thus neutralizing antibodies were not directly assessed in our study on unvaccinated Bangladeshi adults with natural SARS CoV-2 infection. Although we assume the neutralizing antibodies are in part represented by the anti-S1 protein IgG binding activity in the Euroimmun ELISA, we cannot distinguish between anti-S-protein IgG directed against the delta variant versus other variants.

Our finding of anti-N-protein IgG positively correlated with anti-S-protein IgG con-firms the positive correlation previously reported for unvaccinated natural SARS CoV-2 infection [42] and are consistent with the anti-N-protein IgG levels in Novalisa previously reported among Bangladeshi COVID-19 patients [43]. During the first 21 days POCS, anti-N protein IgG levels are typically higher than the anti-S-protein IgG levels [42]. Anti-S protein IgG reach peak levels at 21 days and remain positive beyond 180 days POCS [39]. This trajectory of antibody response may not necessarily apply for asymptomatic and mild cases of COVID-19 which do not develop nucleocapsid antibodies or for populations having SARS CoV-2 reinfection [39,44]. The S1-protein IgG Euroimmun ELISA reported higher rates of sample positivity compared with the S protein IgG Novalisa or the S-protein IgG automated immunoassays of Roche, Diasorin and Abbot [45]. In this respect, we are cautious about interpreting the S-protein IgG levels and acknowledge the higher sensitivity of Euroimmun ELISA for anti-S-protein IgG detection compared with the ELISA or the automated immunoassays of other manufacturers.

## 5. Conclusions

The emergence of SARS CoV-2 variants in unvaccinated populations of low-income countries is a complex process dependent on the coronvavirus-2 molecular epidemiology and specific socioeconomic conditions. In many Bangladeshi households, the extended families of grandparents, parents, and children often live together, which likely raises the risk of viral transmission. Our findings on the Bangladeshi SARS CoV-2 delta variants underscore the potential risk of under-vaccinated and under-monitored populations of low-income countries regarding local public health and the global cooperative response against pandemics.

## Figures and Tables

**Table 1:** Patient characteristics. The patient characteristics in four groups of SARS-CoV-2 variants were compared. The *p*-values are either the rank sum of median differences between group 1 versus group 2 in the Mann–Whitney independent test or the difference in proportions between group 1 versus group 2 in the Chi-square analysis (b). Time points (A) = day 2 or 3 and (B) = day 13 or 14 post onset of COVID-19 symptoms (POCS). NR = not relevant.

Characteristics	SARS-CoV-2 Alpha, Beta Group 1		SARS-CoV-2 Delta Group 2		SARS-CoV-2 Delta Plus Group 3		SARS-CoV-2 B.1.1.318, Eta Group 4		*p* Value 1 vs. 2
Number of cases, N	5		24		3		4		NR
SARS CoV-2 Variant, %	14		67		8		11		0.03
Age as a median (range), years	45 (30–55)		48 (29–87)		44 (32–55)		40 (28–52)		0.39 (b)
Gender, M/F	2/3		17/7		0/3		2/2		0.2
Hospitalized, n (%)	5 (100)		23 (96)		2 (67)		4 (100)		0.65
Oxygen support, n (%)	1 (20)		11 (46)		0 (0)		3 (75)		0.29
Difficulty breathing, n (%)	1 (20)		22 (46)		0 (0)		3 (75)		0.29
Days post onset of COVID-19 symptoms as a median (range)	3 (2–4)		2 (1–3)		2 (2–3)		2 (2–3)		0.65 (b)
COVID-19 symptoms									
Fever, n (%)	5 (100)		23 (96)		3 (100)		3 (75)		0.9
Cough, n (%)	2 (40)		17 (71)		0 (0)		4 (100)		0.19
Shortness of breath, n (%)	1 (20)		11 (46)		1 (33)		2 (50)		0.29
Loss of taste or smell, n (%)	4 (80)		6 (25)		2 (67)		0 (0)		0.02
Muscle or body pain, or headache	0 (0)		3 (13)		0 (0)		2 (67)		0.4
Viral RNA Cp median (95% CI)	31 (26–32)		24 (19–27)		27 (23–31)		24 (19–30)		0.049 (b)
Number of cases, N	5 (A)	5 (B)	24 (A)	17 (B)	3 (A)	3 (B)	4 (A)	4 (B)	NR
Antibody levels mean OD_450–620nm_ (SD)									
Anti-S-protein IgA	3.7 (2)	4 (1)	2.8 (1.8)	2.5 (1.75)	3.4 (2.3)	3 (1.8)	1.4 (0.25)	2.3 (1.7)	0.37 (b)
Anti-S-protein IgG	1.9 (1)	2 (0.05)	1.96 (1)	2 (0.6)	2.3 (0.2)	1.9 (0.3)	0.65 (0.4)	1.7 (0.7)	0.9 (b)
Anti-N-protein IgG	1.8 (1)	1.9 (0.6)	1.6 (0.9)	1.6 (0.7)	1.3 (0.3)	1.3 (0.4)	0.9 (0.6)	1.6 (1)	0.73 (b)

**Table 2:** Sequence analysis of SARS-CoV-2 lineages and variants. Five mutant sequences were identified and assigned GISAID accession numbers. The rare amino acid (a.a.) mutations in two sequences and the single nucleotide substitutions without a.a. substitutions that affected the melting curve in VirSNiP SARS-CoV-2 typing of the other three sequences are highlighted in red.

Sample Nr	PANGO Lineage	Variant	a.a. Substitution, Sequenced Spike-Protein Fragment	Nucleotide Substitution without a.a. Substitution	GISAID Accession-ID
09	B.1.1.318		T95I, del144, E484K, D614G, P681H	C21846T, del21994-21996, G23012A, T23287C, A23403G, C23604A	EPI_ISL_2956623
16	B.1.1.318		T95I, del144, E484K, D614G, P681H	C21846T, del21994-21996, G23012A, T23287C, A23403G, C23604A	EPI_ISL_2956624
28	B.1.617.2	Delta	G142D, E156G, del157/158, L452R, T478K, D614G, Q677H, P681R	G21987A, del22029-22034, T22917G, C22995A, A23403G, G23593T, C23604G	EPI_ISL_2956625
31	B.1.525	Eta	Q52R, A67V, del69/70, del144, E484K, D614G, Q677H, V687L	A21717G, C21762T, del21766-21771, del21994-21996, G23012A, A23403G, G23593C, G23621T	EPI_ISL_2956628
39	B.1.617.2	Delta	G142D, E156G, del157/158, L452R, T478K, D614G, P681R	G21987A, del22029-22034, T22917G, C22995A, C23029T, A23403G, C23604G	EPI_ISL_2956629

## Data Availability

All of the sequencing data and information for this study are available in the GISAID. The accession numbers are provided.

## Supplementary Materials

The following are available online at https://www.mdpi.com/article/10.3390/v13112310/s1, Table S1: PCR primer pairs for sequencing of the SARS CoV-2spike protein.

## References

[1] John Hopkins University of Medicine Coronavirus. https://coronavirus.jhu.edu/.

[2] European Medical Agency EMA. https://www.ema.europa.eu/en.

[3] Ball P. (2021). The speedy approach used to tackle SARS-CoV-2 could change the future of vaccine science. Nature.

[4] Thanh T., Andreadakis Z., Kumar A., Gomez-Roman R., Tollefsen S., Saville M., Mayhew S. (2020). The COVID-19 vaccine development landscape. Nat. Rev..

[5] De Oliveira B.R., Da Penha Sobral A.I.G., Marinho M.L., Sobral M.F.F., De Souza Melo A., Duarte B.G. (2021). Determinants of access to the SARS-CoV-2 vaccine: A preliminary approach. Int. J. Equity Health.

[6] Kaim A., Siman-Tov M., Jaffe E., Adini B. (2021). From isolation to containment: Perceived fear of infectivity and protective behavioral changes during the COVID-19 vaccination campaign. Int. J. Environ. Res. Public Health.

[7] Siam H.B., Hasan M., Tashrif S.M., Khan H.R., Raheem E., Hossain M.S. (2021). Insights into the first seven-months of COVID-19 pandemic in Bangladesh: Lessons learned from a high risk country. Heliyon.

[8] Wilson S.L., Wiysonge C. (2020). Social media and vaccine hesitancy. BMJ Glob. Health.

[9] Rella S.A., Kulikova Y.A., Dermitzakis E.T., Kondrashov F.A. (2021). Rates of SARS-CoV-2 transmission and vaccination impact the fate of vaccine-resistant strains. Nat. Sci. Rep..

[10] Kames J., Holcomb D.D., Kimchi O., DiCuccio M., Hamasaki-Katagiri N., Wang T., Komar A.A., Alexaki A., Kimchi-Sarfaty C. (2020). Sequence analysis of SARS-CoV-2 genome reveals features important for vaccine design. Nat. Sci. Rep..

[11] Pritchard E., Matthews P.C., Stoesser N., Eyre D.W., Gethings O., Vihta K.D., Jones J., House T., VanSteenHouse H., Bell I. (2021). Impact of vaccination on new SARS-CoV-2 infections in the United Kingdom. Nat. Med..

[12] Harvey W.T., Carabelli A.M., Jackson B., Gupta R.K., Tho mson E.C., Harrison E.M., Ludden C., Reeve R., Rambaut A. (2021). COVID-19 Genomics UK (COG-UK) Consortium, Peacock SJ and Robertson DL, SARS-CoV-2 variants, spike mutations and immune escape. Nat. Rev. Microbiol..

[13] Farinholt T., Doddapaneni H., Qin X., Menon V., Meng Q., Metcalf G., Chao H., Gingras M.C., Farinholt P., Agrawal C. (2021). Transmission event of SARS-CoV-2 Delta variant reveals multiple vaccine breakthrough infections. medRxiv.

[14] Elbe S., Buckland-Merrett G. (2017). Data, disease and diplomacy: GISAID’s innovative contribution to global health. Glob. Chall..

[15] Yoo H.M., Kim I.H., Kim S. (2021). Nucleic acid testing of SARS-CoV-2. Int. J. Mol. Sci..

[16] Rambaut A., Holmes E.C., O’Toole Á., Hill V., McCrone J.T., Ruis C., Du Plessis L., Pybus O.G. (2020). A dynamic nomenclature proposal for SARS-CoV-2 lineages to assist genomic epidemiology. Nat. Microbiol..

[17] Rules for the Designation and Naming of Pango Lineages. https://www.pango.network/the-pango-nomenclature-system/statement-of-nomenclature-rules/.

[18] SARS-CoV-2 Variant Classifications and Definitions. https://www.cdc.gov/coronavirus/2019-ncov/variants/variant-info.html.

[19] Investigation of SARS-CoV-2 Variants of Concern: Technical Briefings. https://www.gov.uk/government/publications/investigation-of-novel-sars-cov-2-variant-variant-of-concern-20201201.

[20] Tracking SARS-CoV-2 Variants. https://www.who.int/en/activities/tracking-SARS-CoV-2-variants/.

[21] Barros-Martins J., Hammerschmidt S.I., Cossmann A., Odak I., Stankov M.V., Morillas Ramos G., Dopfer-Jablonka A., Heidemann A., Ritter C., Friedrichsen M. (2021). Immune responses against SARS-CoV-2 variants after heterologous and homologous ChAdOx1 nCoV-19/BNT162b2 vaccination. Nat. Med..

[22] Khoury D.S., Cromer D., Reynaldi A., Schlub T.E., Wheatley A.K., Juno J.A., Subbarao K., Kent S.J., Triccas J.A., Davenport M.P. (2021). Neutralizing antibody levels are highly predictive of immune protection from symptomatic SARS-CoV-2 infection. Nat. Med..

[23] Bernal J.L., Andrews N., Gower C., Gallagher E., Simmons R., Thelwall S., Stowe J., Tessier E., Groves N., Dabrera G. (2021). Effectiveness of Covid-19 Vaccines against the B.1.617.2 (Delta) Variant. N. Engl. J. Med..

[24] Bangladesh Directorate General of Health Services, Ministry of Health and Family Welfare (2021). Coronavirus COVID-19 Dashboard. http://103.247.238.92/webportal/pages/covid19.php.

[25] Saha S., Tanmoy A.M., Hooda Y., Akter A.T., Goswami S., Sium S.M., Islam S.M., Malaker R., Islam S., Rahman H. (2021). COVID-19 rise in Bangladesh correlates with increasing detection of B.1.351 variant. Brit. Med. J. Glob. Health.

[26] Rahman M., Shirin T., Rahman S., Rahman M.M., Hossain M.E., Hossain M.K., Rahman M.Z., El Arifeen S., Ahmed T. (2021). The emergence of SARS-CoV-2 variants in Dhaka city, Bangladesh. Transbound. Emerg. Dis..

[27] Islam A., Sayeed M.A., Rahman M.K., Zamil S., Abedin J., Saha O., Hassan M.M. (2021). Assessment of basic reproduction number (R0), spatial and temporal epidemiological determinants, and genetic characterization of SARS-CoV-2 in Bangladesh. Infect. Genet. Evol..

[28] Hasan M.M., Rocha I.C.N., Ramos K.G., Cedeño T.D.D., Dos Santos Costa A.C., Tsagkaris C., Billah M., Ahmad S., Essar M.Y. (2021). Emergence of highly infectious SARS-CoV-2 variants in Bangladesh: The need for systematic genetic surveillance as a public health strategy. Trop. Med. Health BMC.

[29] Jahan M., Bhattacharjee A., Rahmat R., Islam S.R., Akhter T., Ahammad I., Hossain M.U., Munshi S.U., Salimullahd M. (2021). Genomic surveillance of SARS-CoV-2 viruses collected during the ending phase of the first wave of the COVID-19 pandemic in Bangladesh. Microbiol. Resour. Announc..

[30] Hasan M.M., Das R., Rasheduzzaman M., Hussain M.H., Muzahid N.H., Salauddin A., Rumi M.H., Rashid S.M.M., Siddiki Z., Mannan A. (2021). Global and local mutations in Bangladeshi SARS-CoV-2 genomes. Virus Res..

[31] Hossain M.E., Rahman M.M., Alam M.S., Karim Y., Hoque A.F., Rahman S., Rahman M.Z., Rahmana M. (2021). Genome sequence of a SARS-CoV-2 strain from Bangladesh that is nearly identical to United Kingdom SARS-CoV-2 variant B.1.1.7. Microbiol. Resour. Announc..

[32] Sarkar M.M.H., Rabbi M.F.A., Akter S., Banu T.A., Goswami B., Jahan I., Hossain M.S., Osman E., Uzzaman M.S., Habib A. (2021). Genome sequence of a SARS-CoV-2 P.1 variant of concern (20J/501Y.V3) from Bangladesh. Microbiol. Resour. Announc..

[33] Daria A.A., Assaduzzaman M.S., Islam M.R. (2021). Bangladesh reported delta variant of coronavirus among its citizen: Actionable items to tackle the potential massive third wave. Infect. Prev. Pract..

[34] Das A., Khurshid S., Ferdausi A., Nipu E.S., Das A., Ahmed F.F. (2021). Molecular insight into the genomic variation of SARS-CoV-2 strains from current outbreak. Comput. Biol. Chem..

[35] Nagy A., Basiouni S., Prvin R., Hafez H., Shehata A.A. (2021). Evolutionary insights into the furin cleavage sites of SARS-CoV-2 variants from humans and animals. Arch. Virol..

[36] Grabowski F., Preibisch G., Gizinaki S., Kochanczyk M., Lipniack T. (2021). SARS-CoV-2 variant of concern 202012/01 has about twofold replicative advantage and acquires concerning mutations. Viruses.

[37] Grabowski F., Preibisch G., Gizinski S., Kochanczyk M., Lipniack T. (2021). Immune evasion of SARS-CoV-2 emerging variants: What have we learnt so far?. Viruses.

[38] Tegally H., Ramuth M., Amoaka D., Scheepers C., Wilkinson E., Giovanetti M., Lessells R.J., Giandhari J., Ismail A., Martin D. (2021). Genomic epidemiology of SARS-CoV-2 in Mauritius reveals a new wave of infections dominated by the B.1.1.318, a variant under investigation. medRxiv.

[39] Wei J., Matthews P.C., Stoesser N., Maddox T., Lorenzi L., Studley R., Bell J.I., Newton J.N., Farrar J., Diamond I. (2021). Anti-spike antibody response to natural SARS-CoV-2 infection in the general population. Nat. Commun..

[40] Kumar S., Stecher G., Li M., Knyaz C., Tamura K. (2018). MEGA X: Molecular evolutionary genetics analysis across computing platforms. Mol. Biol. Evol..

[41] Dolscheid-Pommerich R., Bartok E., Renn M., Kümmerer B.M., Schulte B., Schmithausen R.M., Stoffel-Wagner B., Streeck H., Saschenbrecker S., Steinhagen K. (2021). Correlation between a quantitative SARS CoV-2 IgG ELISA and neutralization activity. J. Med. Virol..

[42] Brochot E., Demey B., Touzé A., Belouzard S., Dubuisson J., Schmit J.L., Duverlie G., Francois C., Castelain S., Helle F. (2020). Anti-spike, anti-nucleocapsid and neutralizing antibodies in SARS-CoV-2 in patients and asymptomatic individuals. Front. Microbiol..

[43] Lytton S., Yeasmin M., Ghosh A., Bulbul R.H., Molla M.A., Herr M., Duchmann H., Sharif M., Nafisa T., Amin R. (2021). Detection of anti-nucleocapsid antibody in COVID-19 patients in Bangladesh is not correlated with previous dengue infection. Pathogens.

[44] Egbert E.R., Xiao S., Colantuoni E., Caturegli P., Gadala A., Milstone A.M., Debes A.K. (2021). Durability of spike immunoglobin G antibodies to SARS-CoV-2 among health care workers with prior infection. JAMA.

[45] Swadźba J., Bednarczyk M., Anyszek T., Kozlowska D., Panek A., Martin E. (2021). The real life performance of 7 automated anti-SARS-CoV-2 IgG and IgM/IgA immunoassays. Pract. Lab. Med..

