# Supplementary material for: Molecular and Serological Characterization of the SARS-CoV-2 Delta Variant in Bangladesh in 2021
Source: Viruses. 2021 Nov 19;13(11):2310. doi: 10.3390/v13112310 (PMC8623815; doi:10.3390/v13112310)
Supplement: Supplementary file 1 [file viruses-13-02310-s001.zip › viruses-1428953-supplementary.pdf]

viruses

MDPI

Table S1. PCR primer pairs for sequencing of the SARS CoV-2spike protein. The six forward (F) and reverse (R) primers are listed with their position in SARS CoV-2 genomic sequence and the corresponding amplicon fragment size.

, Primer Name	Sequence (5´- 3´)	Position NC_045512	PCR Fragment Size
Spike F1	TgCCACTAgTCTCTAgTCAgTgTgT	21585-21609	454 bp
Spike R1	AACTCTgAACTCACTTTCCATCCA	22039-22016	
Spike F2	gAAgACCCAgTCCCTACTTATTgT	21898-21921	432 bp
Spike R2	TgAAgAAgAATCACCAggAgTCA	22330-22308	
Spike F3	CAATAggTATTAACATCACTAggTTTCA	22251-22278	501 bp
Spike R3	gCATAgACATTAgTAAAgCAgAgATCA	22752-22726	
Spike F4	TATTCTgTCCTATATAATTCCgCATCA	22655-22681	479 bp
Spike R4	AACAgTTgCTggTgCATgTAgA	23134-23113	
Spike F5	TTTCCTTTACAATCATATggTTTCCA	23030-23050	438 bp
Spike R5	AAACACgCCAAgTAggAgTAA	23468-23448	
Spike F6	CAAATACTTCTAACCAggTTgCTg	23367-23390	411 bp
Spike R6	gTACAATCTACTgATgTCTTggTCATAgA	23778-23750	
